# 506. Outpatient Bamlanivimab, Casirivimab and Imdevimab for COVID-19: Single Center Feasibility Analysis

**DOI:** 10.1093/ofid/ofab466.705

**Published:** 2021-12-04

**Authors:** Vahini Chundi, Jennifer J Wenner, Christopher Scheib, Yatin Patel, Cynthia snider, Jeffrey C Hatcher, Douglas B McQuaid

**Affiliations:** Cone Health, Greensboro, North Carolina

## Abstract

**Background:**

Monoclonal Antibodies directed at the spike protein of SARS-COV-2 are approved by the FDA for Emergency Use for outpatients with COVID- 19 who are at risk for severe complications. Here we present a single center experience using Bamlanivimab and Casirivimab/Imdevimab to prevent hospitalizations due to SARS-COV-2.

**Methods:**

Adult patients who tested positive for SARS-COV-2 in our health system were offered outpatient monoclonal antibody infusion if: (1) testing was done within the previous 7 days, (2) the patient had fewer than 10 days of symptoms, (3) the patient was not currently hospitalized, and (4) met at least 1 of 8 criteria in the FDA EUA Fact Sheet for Bamlanivimab and Casirivimab/Imdevimab. Patients who met the criteria were offered the monoclonal antibody available at time of infusion. Those who declined antibody infusion were used as potential controls. The primary outcome was the discrepancy in hospitalization rates at 14-days past the infusion date for patients receiving the monoclonal antibody regimen versus 14-days past when those in control group would have been scheduled for infusion had they accepted. Secondary outcomes included emergency room visits, duration of hospitalization, and Intensive Care Unit stays. Coarsened exact matching (CEM) was used to obtain balance between treatment and control groups. A logistic regression model measured statistical differences between the groups.

**Results:**

Between November 23, 2021 and February 8, 2021, 5567 patients were offered a monoclonal antibody infusion. A total of 894 patients completed infusion who were able to be matched with patients in the control group. Patients who received the infusion were statistically less likely to be hospitalized than those who did not receive the infusion (2.68% vs 6.70%, p< 0.001).

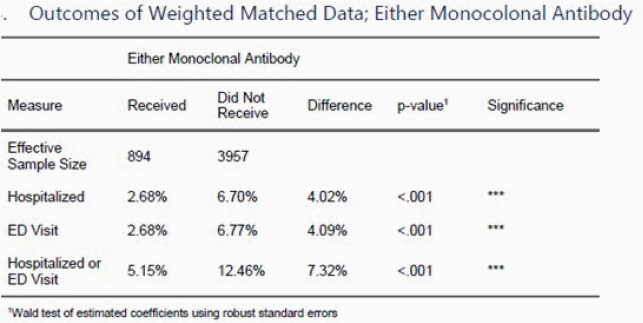

**Conclusion:**

This feasibility study shows reduction in hospitalization in patients who received monoclonal antibody versus standard care. It provides real-world information regarding using monoclonal antibodies as a tertiary prevention strategy to limit the progression of SARS-CoV2 infections, which will lead to improved clinical outcomes and decreased healthcare costs.

**Disclosures:**

**All Authors**: No reported disclosures

